# Image and data processing algorithms for identifying cell-bound membrane vesicle trajectories and movement information

**DOI:** 10.1016/j.dib.2018.12.076

**Published:** 2018-12-28

**Authors:** Ye Xu, Wendiao Zhang, Yong Chen, Wenzhe Shan

**Affiliations:** a Nanoscale Science and Technology Laboratory, Institute for Advanced Study, Nanchang University, Nanchang, Jiangxi 330031, PR China; b Department of Civil Engineering, Nanchang University, Nanchang, Jiangxi 330031, PR China

## Abstract

This DIB article provides details about the trajectory identification and data processing algorithms used in the article “Dynamic single-vesicle tracking of cell-bound membrane vesicles on resting, activated, and cytoskeleton-disrupted cells” (Zhang et al.) [1]. The algorithm identifies vesicles on cell membranes from series of undyed grayscale images captured by the confocal microscope based on contrast differences and then trajectories of vesicles are obtained by analyzing their positions in consecutive images. Once the trajectories have been obtained, other quantitative movement information, such as moving speed, direction and acceleration, are derived by standard dynamic relations.

## Specifications table

**Table:** 

Subject area	*Biology, Image processing*
More specific subject area	*Point-like feature identification and trajectory tracing*
Type of data	*Images, Computer algorithms, Source code*
How data was acquired	*Confocal Microscope, In-house MATLAB program*
Data format	*Analyzed*
Experimental factors	*The image processing algorithms introduced are implemented in MATLAB. The raw images processed by the program are undyed grayscale images captured by the Confocal microscope.*
Experimental features	*In-house developed image processing algorithm for identifying positions and trajectories of undyed point-like features from series of raw images, as well as obtaining their moving status (velocity, moving range, etc.).*
Data source location	*Nanchang, Jiangxi Province, China*
Data accessibility	*Algorithms, equations and codes are presented in this article.*
Related research article	*W. Zhang, Y. Xu, G. Chen, K. Wang, W. Shan, Y. Chen, Dynamic single-vesicle tracking of cell-bound membrane vesicles on resting, activated, and cytoskeleton-disrupted cells, BBA-Biomembranes, 1861(1):26–33*[1].

## Value of the data

• Algorithms and source code are provided and can be used for identifying multiple point-like features on grayscale raw images (dyeing is not required for the experiment).
• Algorithms and source code are provided and can be used for identifying the moving trajectories of multiple point-like features from series of grayscale raw images (dyeing is not required for the experiment).
• Algorithms and source code are provided and can be used to calculate the movement information, e.g. velocity and moving range, from identified trajectories of moving point-like features.

## 1 Data

Three types of data are used in the article [1] for analyzing the moving status of vesicles on cell membranes: mean velocity, moving range (displacement) and population of vesicles on different cell samples. These three types of data are obtained from their positions and trajectories (**TRJ**) extracted from series of raw images captured by the confocal microscope (**CM**). This report presents algorithmic and implementation details about our **in-house developed programs** using MATLAB, which is used for extracting the TRJs from unmarked raw images, as well as obtaining the abovementioned data from the TRJs. The following key algorithms are explained in the next section: vesicle-identification, trajectory-tracing and postprocess for additional movement information. Source codes of corresponding algorithms are provided (code 1–code 14), as well.

## 2 Experimental design, materials and methods

### 2.1 Algorithm for the vesicle-identification

First, the pixel data are extracted from raw images that are in the RGB format, where each pixel contains three color-data [red, green, blue], ranging from 0 to 254. Since images captured by the CM are stored in the grayscale-style, the three color-data are identical for each pixel and we can take an arbitrary one for the image processing. The source code for this step is given in Code 1.



After the raw data extraction, the so-called Laplacian of Gaussian (**LOG**) algorithm, which is a standard image-processing algorithm for boundary identification, is used to isolate pixel groups corresponding to vesicles. The raw image data corresponds to redness (brightness in red) of each pixel and the LOG values of pixels corresponding to vesicles will be significantly higher than the rest of the image. Therefore, a lower bound can be used to isolate the pixels corresponding to vesicles from the rest of the image. Such lower bound is obtained by comparing the filtered results with human-eye observations in a try-and-error manner. The filtered image data is than normalized back into the grayscale format, so that each pixel will be assigned a value ranging from 0 to 1.After the normalization, the pixel data is then converted into the black-and-white style so that those corresponding to vesicles will have the value of 1 and the rest pixels will have the value of 0, ready for the next step. The additional lower-bound filter mentioned above also guarantees the boundary for vesicles which are close to each other will have their boundaries set to zero. The source code for this step is given in Code 2.



After applying the LOG filter and converting the pixel data into the black-and-white style, the filtered pixel data will look like a group of white blobs with approximately the same size corresponding to the vesicles in a black background, a recursive algorithm which checks the neighboring pixels is then applied to identify and store them in a more organized format and their centers are then computed and used as the positions of vesicles which will be used later for the trajectory-identification and postprocess. In addition to center coordinates, the size of each blob (number of pixels) will also be counted so that and additional lower bound can be defined to filter out noise blobs whose size are significantly smaller than actual vesicles. Such lower bound should be approximately the number of pixels of the smallest vesicle, which is identified by human-eye observations. The source code for this step is given in Code 3.



With all the steps above, the vesicle positions in all the images are then ready to be identified, or more specifically, by applying step 2 and step 3 to the pixel data read from all raw images in step 1, whose details are given in Code 4.



### 2.2 Algorithm for trajectory tracing

From human-eye observations, the number of vesicles in each image can be different and their movements can be categorized into the following five types:

1. Move and oscillate within a small region;
2. Move in a very large region with large speed;
3. Some vesicles vanish;
4. Multiple vesicles merge into a single one;
5. Some new vesicles emerge.

In accordance, four types of trajectories must be allowed for the tracing algorithm:

• **Complete trajectories**: traced through the entire series of images
• **Incomplete trajectories**: only traced in a portion of consecutive images
• **Branched trajectories**: a single trajectory expands into multiple branches at certain step
• **Merged trajectories**: multiple trajectories merge into one at certain step.

Therefore, the number of trajectories is most likely NOT equal to the number of vesicles and a relatively complicated data structure is defined for storing and manipulating the trajectory data. In this DIB, it is given the name **TRJ**, which is a compound data structure containing the following members:

• **TRJ.counter**: the number of trajectories stored;
• **TRJ.pos**: array of pointers that access the data of each trajectory;
• **TRJ.nframe**: number of images used for tracing trajectories;
• **TRJ.dat**: group of trajectory data. The information of each trajectory in stored in a three-column matrix as: [**iframe, pid1, pid2**], where **iframe** is the index of the current image, **pid1** is the index of vesicle in the previous image and **pid2** is the index of vesicle in the current image. Such type of data structure is used because vesicles in each image are identified by the same algorithm mentioned above and therefore vesicles with same indices in different images are most likely not the same.

**Fig. 1:**
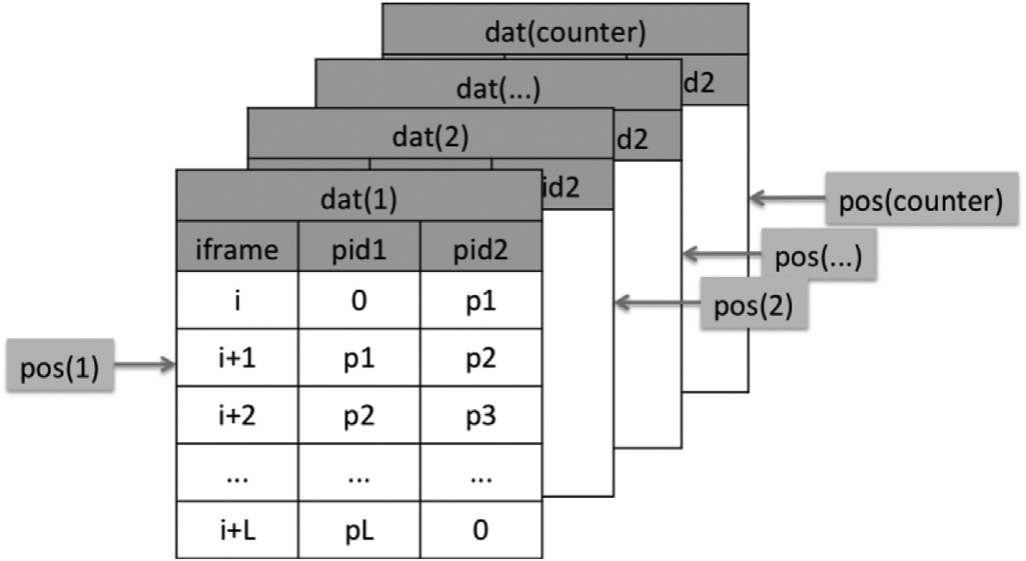
Schematic illustration of the compound data structure storing trajectories (TRJ).

The schematic of the TRJ structure is illustrated in Fig. 1.

**Fig. 2:**
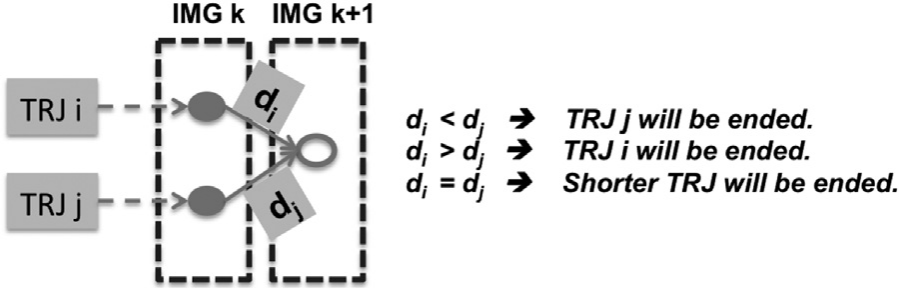
Merging of trajectories.

**For the tracing process**, the following methods are defined for TRJ:

1. **Initialization**: initialize the data structure and allocate memory.
2. **New**: add a new trajectory.
3. **Tails**: get the tails of all live trajectories, which are equal to the locations of their local pointers during the tracing process. A live trajectory means its end has not been identified yet, and the judging criteria is (pid2 > 0 in Fig. 1) for its current segment, so that it is considered as ‘alive’ for the tracing process. Within this method, trajectories with identical tails will be identified, which corresponds to the merging scenario mentioned above, and those with the same tail will be compared by their lengths, where only the longest will be kept alive while the rest will be killed (considered as merged), as illustrated in Fig. 2.

The source code for the ‘Tails’ method is given in Code 7.

4. **Insertion**: append new segments to live trajectories. If no corresponding live trajectories are found for certain new segments, new trajectories will be created, using them as the head segments. The criteria for identifying segments of new trajectories is: pid1 == 0 and pid2 > 0, as shown in Fig. 1, and the source code for this method is given in Code 8.
5. **Coordinates Extraction**: extract coordinates by vesicle indices and image indices. Since only vesicle indices and image indices are stored in the TRJ data structure, such coordinate extraction method must be defined to get the actual coordinates of vesicles along their trajectories, using the results obtained from the vesicle-identification process. The source code for this method is given in Code 9.

Limited by experiment conditions, vesicles cannot be individually tagged and traced by techniques other than visually comparing their positions in consecutive images. Therefore, the **criteria for identifying two vesicles in two consecutive images belonging to the same trajectory** are:

• These two vesicles are closest to each other than the rest;
• Their distance should be smaller than a given cutoff distance that corresponds to the maximum moving distance of a vesicle for the time interval between two consecutive images.

**Fig. 3:**
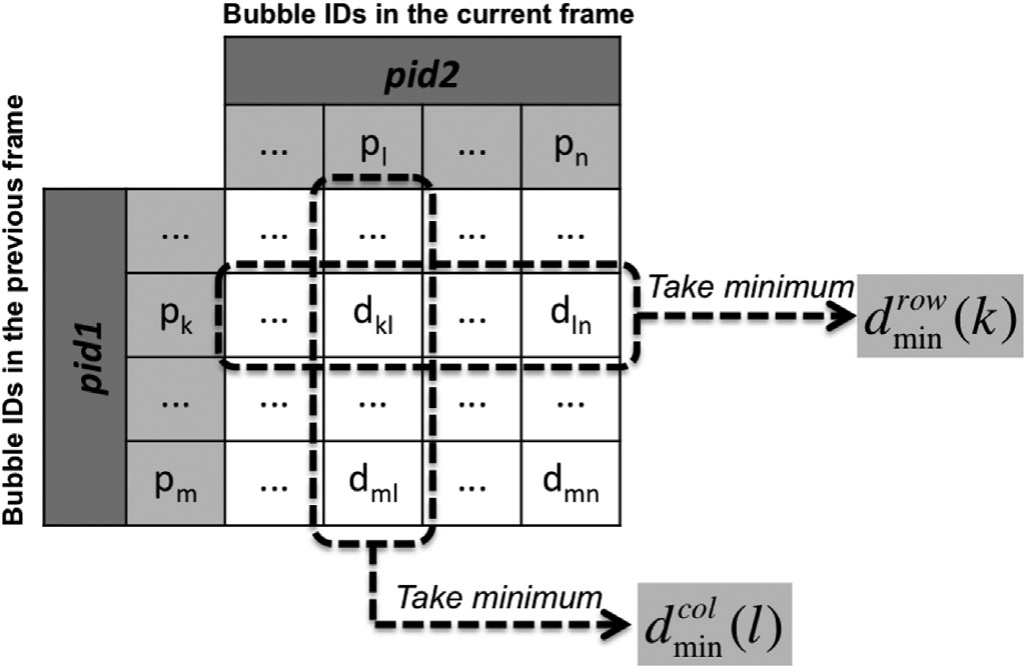
Distance matrix used for determining closest vesicles/bubbles in two consecutive images.

**Fig. 4:**
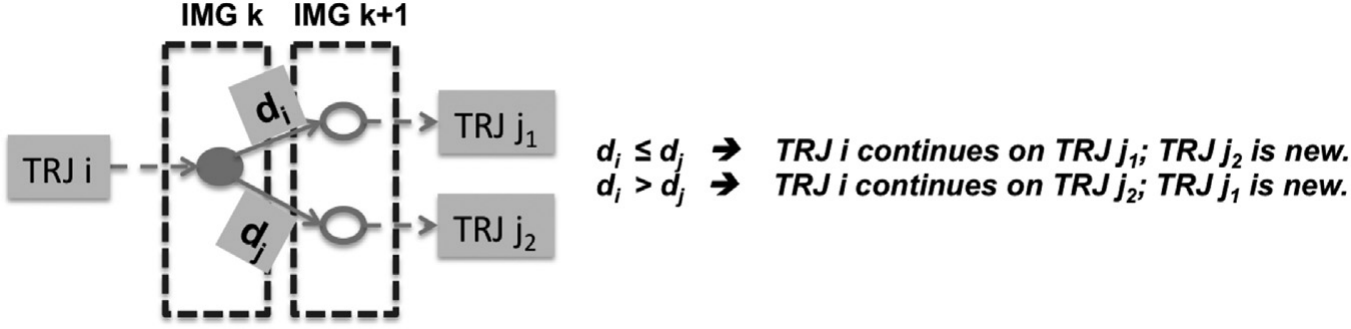
Branching of a trajectory.

The closest vesicles in two consecutive images are determined by using the **distance matrix** shown in Fig. 3, where distances between all pairs of vesicles in two images are computed and stored. Minimum values are taken for each row and each column and the corresponding column index and row index are stored as well. Taking the **row minimum** is equivalent to finding the closest vesicles in the current frame to the previous frame, while taking the **column minimum** determines the closest vesicles in the previous frame to the current frame. If the obtained minimum distance is less than the cutoff distance, then it is considered as a valid value. The results can be categorized into the following five types:

1. **Regular movement:** A vesicle in the current frame is the closest neighbor to a *unique* vesicle in the previous frame and vise versa. In the distance matrix, a unique row minimum can be found at the corresponding column;
2. **Merging**: A vesicle in the current frame is the closest neighbor to *more than one vesicles* in the previous frame (as illustrated in Fig. 2); In the distance matrix, more than one row minimums will be found at the corresponding column;
3. **Branching**: A vesicle in the previous frame is the closest neighbor to *more than one* vesicles in the current frame (as illustrated in Fig. 4); In the distance matrix, more than one column minimums will be found at the corresponding row;

4. **End of a trajectory**: For a vesicle in the previous frame, no vesicle in the current frame can be considered as a valid neighbor (distance too large).
5. **Begin of a new trajectory**: For a vesicle in the current frame, no vesicle in the previous frame can be considered as a valid neighbor (distance too large).

The source code for identifying the connectivity of vesicles identified in two consecutive images is given in Code 10.



Based on human-eye observations of raw images, the speed of vesicles can vary significantly. Some vesicles can move much faster than others, while the speed of the same vesicle can also sometimes change significantly during a time period. Therefore, a single cutoff distance for the connectivity search can be inconvenient. A small cutoff distance can improve the accuracy of the tracing algorithm for slow vesicles, but very likely miss many fast ones, while a large cutoff distance will decrease the accuracy of the algorithm for vesicles close to each other (mixing-up). Hence, an improved algorithm based on Code 10 is used for searching the connectivity between two groups of points. For the new algorithm, multiple cutoff distances ranging from a minimum value to a maximum value are used, and the implementation is given in Code 11.



Trajectories of vesicles can then be traced using the methods introduced above (Code 5–Code 9), as well as the connectivity search algorithms (Code 10–Code 11), and stored in the TRJ data structure. The implementation for the combined tracing algorithm is given in Code 12.



To improve the efficiency of the program, the TRJ data needs to be initialized with an overestimated number of possible trajectories. Therefore, a trimming function should be used after the tracing process to free unused memory, whose implementation is given in Code 13.



### 2.3 Postprocess for trajectories

**Fig. 5:**
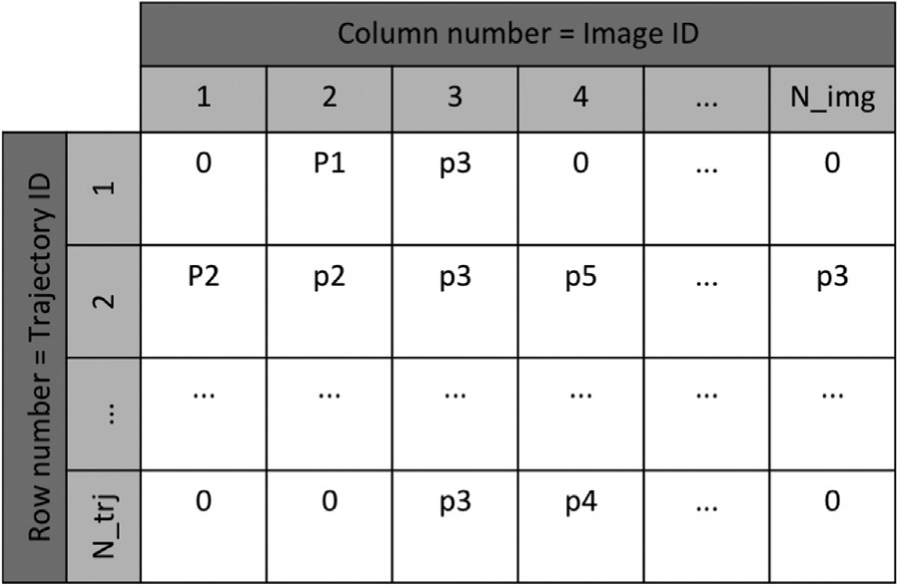
Trajectory matrix used for the postprocess, where elements are indices of identified vesicles in each image whose index is the column number, while the row number is the index of trajectories. A trajectory started with the first non-zero vesicle index and ended with the last non-zero index. In the above example, the 1st and 2nd trajectories merged at the 3^rd^ frame, while the 2nd trajectory branched to the last trajectory at the 4th frame.



The postprocess for trajectories includes visualization and calculation of extra information such as velocity, speed, moving range (displacement), etc. from the obtained vesicle positions and trajectory data. The design of the TRJ data structure illustrated in Fig. 1 is for the convenience of the tracing process, but its direct usage for the postprocess is not efficient. Instead, we designed a **trajectory matrix** that can be extracted from the TRJ data after the identification process for the postprocess. The schematic of the matrix structure is shown in Fig. 5. And the MATLAB code for extracting the matrix is given in Code 14.

The sequential coordinates of vesicles corresponding to each trajectory can be easily obtained by using the trajectory matrix, together with the coordinate generation code given by Code 9. Then the velocity, speed and moving range (displacement), as well as other kinds of movement information can be readily derived. In addition, by counting the number of non-zero elements in each row of the trajectory matrix, the life (how many images a trajectory lasts) of each trajectory can be obtained, which can be used for controlling the quality of trajectories used for calculating statistical information. For example, adding a lower-bound filter to the trajectory life can allow us to exclude short-lived trajectories most likely caused by image noises.

The **velocity** of a vesicle along a trajectory is computed by

$$\overset{\to }{v}\left(n\right)=\{\begin{array}{l} \frac{\overset{\to }{x}\left(n+1\right)-\overset{\to }{x}(n)}{∆t},\;n=1\\ \frac{\overset{\to }{x}\left(n+1\right)-\overset{\to }{x}(n-1)}{2∆t},\;1<n<N\_\mathrm{img}\\ \frac{\overset{\to }{x}\left(n\right)-\overset{\to }{x}(n-1)}{∆t},\;n=N\_\mathrm{img} \end{array}$$

where $\vec{v}\left(n\right)$ is the velocity at the time corresponding to the image of index *n*, $\overset{\to }{x}(n)$ is the vesicle position, ∆t is the time interval between two consecutive images and *N_img* is the number of images. The **speed** is by definition the magnitude of the velocity, namely

$$v\left(n\right)=\left\|\vec{v}\left(n\right)\right\|$$

The **traveling distance** of a vesicle along its trajectory is computed by

$$s\left(n\right)=\{\begin{array}{l} 0,\;n=1\\ \overset{n}{\underset{i=2}{\sum }}\left\|\overset{\to }{x}\left(i\right)-\overset{\to }{x}(i-1)\right\|,\;n\leq N\_\mathrm{img} \end{array}$$

The **moving range (displacement)** of a vesicle along its trajectory is defined by

$$R\left(n\right)=\{\begin{array}{l} 0,\;n=1\\ \max\left\{\left\|\overset{\to }{x}\left(i\right)-\overset{\to }{x}\left(1\right)\right\|\right|1<i\leq n\},\;n\leq N\_\mathrm{img} \end{array}$$

Implementations for calculating the above variables are quite straightforward and are therefore omitted here.
